# Classic Hairy Cell Leukemia With MAP2K1 Mutation: Diagnosis and Targeted Therapy

**DOI:** 10.1002/ajh.70043

**Published:** 2025-08-23

**Authors:** Andrea Duminuco, Alessandra Venanzi, Alessia Santi, Alessandro Mancini, Alessandra Romano, Enrico Tiacci

**Affiliations:** ^1^ Institute of Hematology University and Hospital of Catania Catania Italy; ^2^ Institute of Hematology and Center for Hemato‐Oncology Research, Department of Medicine and Surgery University and Hospital of Perugia Perugia Italy

**Keywords:** hairy cell leukemia, MAP2K1 mutation, neoplasia‐lymphoid leukemias, neoplasia‐pharmacotherapeutics, targeted therapy

Hairy cell leukemia (HCL) is caused by the kinase‐activating BRAF‐V600E mutation in > 95% of patients, making this disease sensitive to oral BRAF inhibitors [1, 2, 3]. Rare HCL cases lacking BRAF‐V600E have been recently described that respond suboptimally to standard chemotherapy with purine analogs and harbor alternative, potentially targetable, kinase‐activating short deletions of BRAF or missense mutations of MAP2K1/MEK1, the kinase phosphorylated by BRAF within the RAF → MEK → ERK signaling pathway [4, 5].

Here, we describe a 60‐year‐old male with HCL who had received multiple treatments over the previous 23 years, that is splenectomy and six subsequent lines of chemo‐immunotherapy, with refractoriness to the last one (pentostatin plus rituximab) resulting in persistent cytopenias (hemoglobin 5.7 g/dL, platelets 27 000/mm^3^; neutrophils 1190/mm^3^). This case had clinico‐pathological features typical of classic HCL, including “fried‐egg” histological pattern (Figure 1A) with marked reticulin fibrosis (not shown), as well as circulating leukemic cells with circumferential hairy projections and inconspicuous nucleoli (Figure 1B). However, leukemic cells lacked BRAF‐V600E (Figure 1C), yet showed prominent ERK phosphorylation (Figure 1D) that was due to a clonal activating Q56P mutation of MAP2K1/MEK1 identified upon next‐generation sequencing. Indeed, in vitro treatment of patients' leukemic cells with the MEK inhibitor cobimetinib strongly dephosphorylated ERK and elicited marked apoptosis (not shown).

**FIGURE 1:**
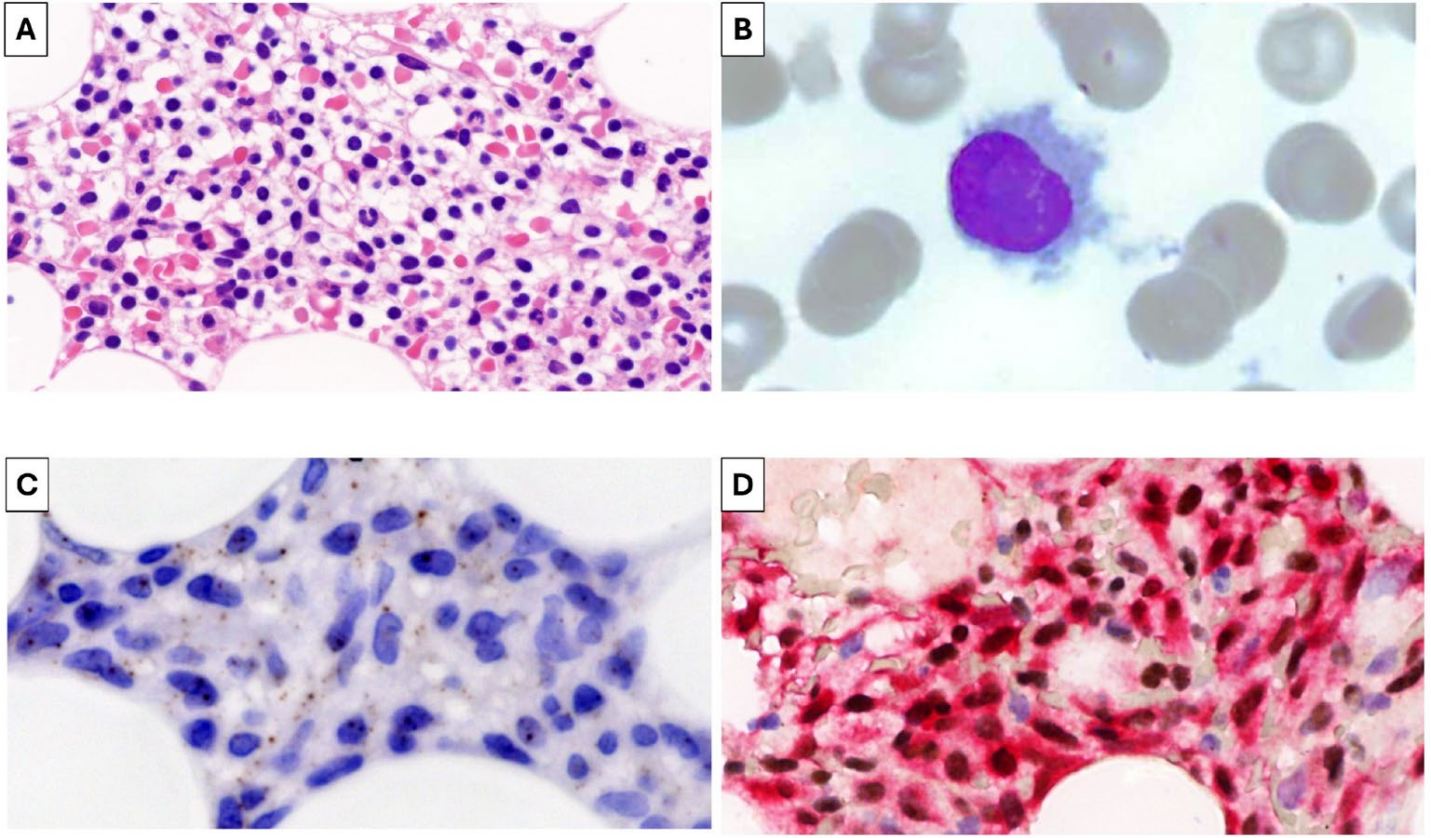
(A) Bone marrow biopsy stained with hematoxylin/eosin and showing diffuse infiltration by mature lymphoid cells with round or oval nucleus surrounded by ample clear cytoplasm, giving rise to a “fried egg” appearance. (B) Blood smear stained according to May‐Grünwald‐Giemsa and showing a leukemic cell with indented nucleus, mature chromatin with no nucleoli, wide cytoplasm and thin surface projections circumferentially distributed. (C) Bone marrow biopsy stained with a BRAF‐V600E mutant‐specific monoclonal antibody showing no labeling in the cytoplasm of leukemic cells (while producing a dot‐like unspecific staining in the intercellular space). (D) Bone marrow biopsy showing strong ERK phosphorylation (red cytoplasmic staining) in leukemic cells (dark brown nuclear staining for the B‐cell transcription factor PAX5).

Because the patient had exhausted all standard therapeutic options, before the genetic and functional characterization of the patient's leukemia was complete, he was empirically started on the BRAF inhibitor vemurafenib (960 mg twice daily for 8 days) plus rituximab 375 mg/sqm (for 1 dose), but was then quickly switched to oral cobimetinib (60 mg daily) for 4 cycles (each comprising 21 days of dosing followed by 7 days of rest), with resolution of cytopenias and achievement of a complete remission immunohistochemically confirmed through a bone marrow biopsy. Since recurrent grade‐2 cutaneous rash due to cobimetinib precluded long‐term continuous treatment with the drug, this response was consolidated with an additional two cycles of cobimetinib (mostly at a reduced dose of 40 mg daily) combined with rituximab (375 mg/sqm intravenously) for eight doses (3 during cobimetinib therapy and 5 thereafter). A complete remission was re‐confirmed at the end of such treatment, including clearing of the MAP2K1/MEK1 mutation, and progression‐free survival was 15 months from the start of treatment.

MAP2K1 mutations were first identified in HCL‐variant [6], a distinct disease that has a worse prognosis than classic HCL [7], and in a patient with relapsed HCL‐variant harboring mutant MAP2K1 (K57N) treatment with the MEK inhibitor trametinib was only modestly active (stable disease after 6 cycles) [8]. The current case highlights the importance of correctly diagnosing classic HCL even in the absence of its hallmark BRAF‐V600E mutation and of searching for alternative, targetable kinase mutations to optimally guide patient management.

## Ethics Statement

This report was prepared in accordance with the Declaration of Helsinki. Written informed consent was obtained from the patient. All identifying details have been omitted or anonymized to protect patient confidentiality.

## Conflicts of Interest

The authors declare no conflicts of interest.
